# Knowledge and Utilization of Iodized Salt and Its Associated Factors at Household Level in Mecha District, Northwest Ethiopia

**DOI:** 10.1155/2019/9763830

**Published:** 2019-03-28

**Authors:** Walleligne Beyene Tariku, Amare Lisanu Mazengia

**Affiliations:** ^1^CDC HIV/AIDS Surge Strategy, Amhara Regional Health Bureau, Bahirdar, Ethiopia; ^2^College of Health Sciences, Salale University, Fiche, Ethiopia

## Abstract

**Background:**

Iodine is one of the essential elements that enables the thyroid gland to produce thyroid hormones, which is vital for growth and development of the brain and central nervous system. More than two billion individuals worldwide live in iodine-deficient areas.

**Objectives:**

The aim of this study was to assess knowledge and utilization of iodized salt at the household level and associated factors in the Mecha district, Northwest Ethiopia.

**Methods:**

A community-based cross-sectional study was conducted from March 10 to April 10, 2018. Data were collected using a pretested and structured questionnaire by a face-to-face interview technique. The use of iodized salt at the household level was tested with the iodine rapid test kit. Data were checked, coded, and entered to EPi Info version 3.5.1 and were exported to SPSS (Statistical Package for Social Science) version 20 for analysis.

**Result:**

A total of 700 head of households were included in the study, of which 639 (91.3%) were females. The overall prevalence of knowledge was 201 (28.7%). Availability of adequately iodized salt was 443 (63.3%). The proportion of proper utilization of adequately iodized salt at the household level was 180 (25.7%). Occupational status, educational status, and residence were predictors of knowledge on the use of iodized salt. Educational status, packaging, and knowledge of respondents on iodized salt were significant to utilization.

**Conclusion and Recommendation:**

Knowledge, availability, and utilization of adequately iodized salt remain very low in the district. Concerned body should improve awareness and availability of adequately iodized salt and how to utilize it properly.

## 1. Background

Iodine is an essential mineral for normal growth and development of the fetus, infant, and child for the normal physical and mental activity of the adult [1]. It enables the thyroid gland to produce thyroid hormones, which are also vital for growth and development of the brain and central nervous system [2]. Failure to have adequate iodine leads to insufficient production of these hormones, which affect many parts of the body collectively known as the iodine deficiency disorders (IDD). These consequences include mental retardation, goiter, growth retardation, reproductive failure, and increased childhood mortality [3, 4]. Iodine deficiency disorders are the major public health problems in developing countries [4]. WHO estimates around 28.3% of African population have goiter and approximately 25% of the global burden of iodine deficiency as measured by disability-adjusted life years occurs in Africa [5]. In 2006, around 120 world countries have implemented salt iodization programs. Of these countries, 34 countries have eliminated iodine deficiency disorders through universal salt iodization (USI) [6]. USI is a remarkably cost-effective public-health strategy which is on average, and the one-time increase in cost is only 3–5 cents per person per year—a price so low that even consumers in the least developed countries would barely notice it, and it is a major global public health success [7]. WHO/UNICEF/ICCIDD have approved a cut-off point of 20–40 parts per million (ppm) for iodine in salt [8]. This cut of point is aimed at having 90% of households using adequately iodized salt (≥15 ppm iodine) and at using salt iodine testing as an indicator for monitoring progress towards universal salt iodization [1, 9, 10]. Around 70% of households in the world used iodized salt by 2000, compared with less than 20% in 1990 [11]. Throughout the Middle East and North Africa region, 64% of households consume adequately iodized salt, with wide variation between countries [12]. In Ethiopia, household use of iodized salt was extremely low which is 15.4%. In view of the low prevalence of use of iodized salt, the Ethiopian government formulated a national nutrition program which is applied from June 2013 to June 2015. The program includes USI as one strategic objective which aimed to increasing the proportion of households using iodized salt from 15.4% to 95% [13]. According to my latest knowledge, there are no similar studies done in the district. So to know about knowledge, utilization, and associated factors of households on iodized salt, more epidemiological data are necessary to assist policymakers in their efforts. Therefore, this study added some input for the country, especially concerning rural districts.

## 2. Objective of the Study

The study was aimed at assessing knowledge and utilization of iodized salt and its associated factors at the household level in Mecha district, Northwest Ethiopia, from March 10 to April 10, 2018.

## 3. Methods and Materials

### 3.1. Study Design

A community-based cross-sectional study was conducted.

### 3.2. Study Area and Period

Mecha district is one of the 15 districts of the West Gojjam Zone in the Amhara region. The district has 40 rural and 6 urban kebeles. It is situated at an altitude ranging from 1800 m to 2500 m. The district receives an average annual rainfall ranging from 1000 mm to 2000 mm and average daily temperature from 24°C to 27°C. The total population and household of the district were about 370032 and 86053, respectively. Of the total population, about 186866 (50.5%) were males [14]. This study was conducted from March 10 to April 10, 2018.

### 3.3. Source Population and Illegibility Criteria

The source population was head of households' (18 years of age and above) who live in the Mecha district. The study participants were the head of households' (18 years of age and above) who live in the Mecha district in selected kebeles. All individuals who were the head of households and who live in the study area for 6 months or more were included. Subjects who did not prepare their food in the household were excluded.

### 3.4. Sample Size Determination and Sampling Procedure

The sample size was determined by using a single population proportion formula. Assumptions include that, with 95% CI, 5% of margin of error reported prevalence of adequately iodized salt (33%) [15], which gives *n* = 340. Since it was the multistage sampling method, the calculated sample size was multiplied by the design effect of 2. Considering 5% none response rate, the final sample size was *n* = 714. A multistage stratified sampling technique was used with the strata of kebeles which exist in urban and rural. A total of 46 kebeles were in the district, of which 40 kebeles were located in the rural district and the remaining 6 kebeles in urban. From those, 20% of the kebeles (8 kebeles from rural and 1 kebele from urban) were selected by using the simple random sampling technique. Then, the total sample was allocated proportional to the household size of each kebeles. By using the family folder of a health extension worker as the frame, the first household from each kebele was identified through the lottery method. Then, the systematic random sampling technique was applied to identify the next household to be included. Selected head of the households who were not found at home at the time of the first visit were revisited 2 times at different times. When the interviewer failed to get the eligible participant in the second visit, the household was registered as nonresponse.

### 3.5. Variables of the Study

#### 3.5.1. Dependent Variables

Dependent variables are knowledge on iodized salt and utilization of iodized salt.

#### 3.5.2. Independent Variables

Sociodemographic characteristics include age, sex, marital status, educational status, income, employment status, and residency of the head of households of being rural or urban. Salt condition includes packaging type, storage condition of salt, type of salt, checking of iodized salt, source of salt purchasing, method of salt storage, practices if salt is moisturized and has impurities, time of salt addition while cooking foods, and knowledge-assessing variables.

### 3.6. Data Collection and Quality Control Procedures

Data were collected using a structured questionnaire and rapid iodine test kit by face-to-face interview with five trained clinical nurses and supervised by two BSC nurses. The questionnaire was developed in English and then translated into Amharic, and finally, it was retranslated into English by another language translator for checking consistency. Two-day training regarding the objectives of the study and ways of administering the questionnaire was given to the data collectors as well as the supervisors by the investigator. The prepared questionnaire was pretested prior to the actual data collection on 36 participants (5% of sample). Interviewers proceeded from house to house and introduced themselves as well as explained the purpose of the study using specific statements in a standard procedure. Data collectors asked the head of households to bring a teaspoon of salt which was used for cooking, and iodine content was determined by a rapid iodine test kit. Principal investigators and supervisors had checked the accuracy and completeness of daily collected data. Any error in data collection was rectified before proceeding with the next day's data collection activity. The interview was conducted in a place where the head of household feels free to express her/his feelings and ideas. Daily meeting was held among the data collectors, supervisors, and the principal investigator. Problematic issues faced during the interview were discussed, and decisions were made.

### 3.7. Data Processing and Analysis

After data collection, each questionnaire was checked for completeness, then coded, entered into Epinfo (version 3.5.1) and was exported to SPSS (Statistical Package for Social Science) version 20 for analysis. During the analysis, frequencies of different variables were determined. Crude odds ratio with the confidence interval was used to measure the association between selected variables and to see statistical significances. Categorical variables were analyzed by using bivariate and multivariate analysis to assess the relative effect of explanatory variables on dependent variables after adjusting for the effects of other factors (adjusted ORs). *P* value < 0.05 was used as the cut-off point for statistical significances. The result was presented in the form of tables and text using frequencies and summary statistics with percentage to describe the study population in relation to relevant variables.

### 3.8. Ethical Issue

Ethical approval was taken from the Mecha district health and administrative office. Then, the selected adults (18 years of age and above) from each household were informed about the purpose of the study, the importance of their participation, and withdraw at any time, and a written consent was obtained prior to data collection. Privacy and confidentiality of information given by each respondent was kept properly.

### 3.9. Operational Definition

#### 3.9.1. Head of Household

It is defined as the person who is responsible for purchasing and cooking food.

#### 3.9.2. Adequately Iodized Salt

When we test the sample salt with the rapid iodine test kit, if the iodine content of the household salt sample was ≥15 parts per million (ppm), deep blue color change was seen and it is called adequately iodized salt [10].

#### 3.9.3. Proper Utilization of Iodized Salt

For proper utilization of iodized salt, respondents should use adequately iodized salt and add salt after cooking is finished [16].

#### 3.9.4. Improper Utilization of Iodized Salt

When respondents did not use adequately iodized salt or added salt during cooking, it is termed as improper utilization of iodized salt [16].

#### 3.9.5. Good Knowledge

Respondents who answered greater than or equal to the mean of knowledge questions have good knowledge on the following: Have you heard of about iodized salt? Why intake of iodized salt is important? Is there a problem exposing iodized salt to sunlight? What will happen to iodized salt when it exposed to sunlight? Is there a problem putting iodized salt in to moisture area? What will happen to the iodized salt when it exposed to moisture? Is there a problem washing iodized salt with water? What will happen to the iodized salt when it washed through water?

#### 3.9.6. Poor Knowledge

Respondents who answered less than mean of knowledge questions have poor knowledge.

## 4. Results

### 4.1. Sociodemographic Characteristics

A total of 714 head of households were included in the study, and 700 responded to the questioners, with a response rate of 98%. Six hundred thirty-nine (91.3%) of the head of households were females. The mean age of participants was 32.7 ± 11 years. Of the total, 610 (87.1%) participants were rural residents. Four hundred forty-three (63.3%) participants were unable to read and write. Five hundred seventeen participants (73.9%) were married. Two hundred thirty-two (33.1%) participants earned <500 Ethiopia birr per month for their family. In regard to occupation, 520 (74.3%) head of households were housewives (Table 1).

### 4.2. Knowledge on Iodized Salt at Household Level

Regarding knowledge of participants about the usefulness of iodized salt and consequence of IDD, 201 (28.7%) participants had good knowledge. Four hundred seventy-one (67.3%) had not heard about iodized salt. Those who heard about iodized salt from mass media were 124 (17.7%). All of them, 700 (100%), stored salt in a dry place, but on the contrary, majority of the respondents ( 465 (66.4%)) exposed the salt to sunlight while it was moisturized (Table 2).

### 4.3. Availability and Utilization of Adequately Iodized Salt

Availability of adequately iodized salt which showed deep blue (≥15 ppm) was found in 443 (63.3%) of households. Before testing the salt, 494 (70.6%) participants said that they did not know what type of salt they used, and 174 (24.9%) participants said that they have adequately iodized salt for cooking food. One hundred fifty-seven (22.4%) participants checked salt whether it is iodized or not by reading the pack level. Six hundred forty-three (91.9%) participants purchased salt from shops and also 598 (85.4%) participants stored salt in a jar with a cover. One hundred fifty-eight (22.6%) households used packed salt; of these, 121 (76.6%) participants were adequately iodized. After testing the salt sample using rapid test kits, among those who said that they had iodized salt, 132 (75.9%) households have adequately iodized salt. Out of those who said that they did not know whether it is iodized salt or not, 302 (61.1%) of salt samples were adequately iodized. Among all participants, only 180 (25.7%) were properly utilized iodized salt (Table 3).

### 4.4. Factors Associated with Knowledge on the Use of Iodized Salt

Bivariate and multivariate logistic regression were done to assess factors associated with knowledge on the use of iodized salt. In bivariate analysis, variables which had *P* value < 0.2 entered into multivariate logistic regression analysis. Factors which had significant association on bivariate analysis on knowledge were sex of the head of households, age of the head of household, educational status, residence, family income, and occupational status. Among these factors, residence with AOR (95% CI) = 9.1 (3.6, 22.5), educational status with AOR (95% CI) = 15.0 (9.1, 27.7), and occupational status with AOR (95% CI) = 8.8 (3.1, 24.7)) were significantly associated with knowledge on use of iodized salt during multivariate analysis (Table 4).

### 4.5. Factors Associated with Utilization of Iodized Salt

Among factors which were associated with utilization of iodized salt on bivariate analysis, only educational status, packing, and knowledge of respondents on iodized salt use were significantly associated in multivariate analysis. Participants who attended formal education were 2 times more likely to use iodized salt properly than those who did not attend formal education (AOR (95% CI) = 2.2 (1.2, 3.7)). Participants who had good knowledge on iodized salt were 3.8 times more likely to use iodized salt properly than those who had poor knowledge on iodized salt (AOR (95% CI) = 3.8 (2.1, 6.8)). Participants who used packed salt were 3 times more likely to use iodized salt properly than those who used nonpacked salt (AOR (95% CI) = 3.3 (1.9, 5.7)) (Table 5).

## 5. Discussion

In this study, 201 (28.7%) participants had good knowledge on the use of iodized salt which was not far from studies done in Gondar town and Laelay Maychew district, which were 25.2% and 35.8%, respectively [15, 17]. It had also showed similar result with the study done in Iraqi which was 27.1% [18]. However, it was very low as compared to the study done in Ghana which was 90.4% [19]. Even if there was minimal increment in knowledge on the iodized salt use over the years, it was low, suggesting that awareness creation and educational activities on the use of iodized salt were not yet enough mainly in the rural communities.

Knowledge on iodized salt in the current study was influenced by educational status, occupational status, and residence of participants. Thus, participants who live in the urban area were 9 times more likely to be knowledgeable on the use of iodized salt than those who live in the rural area. This finding was similar with the study done in Sudan [20]. The reason behind this might be that participants who live in the urban area were easily accessible to health education and awareness creation which is now broadcasted through EBC by Ethiopian Food, Drugs and Health Administrative Control Authority and to markets of iodized salt.

Participants who attended formal educations were 15 times more likely being knowledgeable on the use of iodized salt than those who did not attend formal education. This finding was also similar with the study done in Sudan and Iraqi which identified education as a predictor variable to know about iodized salt [18, 20]. The reason for this might be the educated head of households might have learnt and read about the importance of iodized salt.

Participants who were merchants and government employees were 8.8 times and 3 times more likely to be knowledgeable on the use of iodized salt than those who were housewives, respectively. This is because participants who were government employees and merchants were easily accessible to health education and awareness creation is now broadcasted through EBC by EFMHCACA to markets of iodized salt.

Although knowledge on use of iodized salt was very low, knowledge by itself does not guarantee for availability and utilization of adequately iodized salt. So this study tried to assess factors associated with utilization. Availability of adequately iodized salt at the household level was 443 (63.3%) which was higher compared to study done in the Gondar town (28.9%), in Benishangul Gumuz Region (20.1%), EPHI (52.5%) and Laelay Maychew district (33%) [15, 17, 21, 22]. The probable reason might be due to difference in geographic locations, time gap between studies, and inaccessibility to awareness creation and health education. Yet the current rate was still considerably below the national goal of 95% coverage [13]. Furthermore, this study was low when compared to studies done in Ghana, India, and Nigeria which were 75.6%, 83.1%, and 97.5%, respectively [19, 23, 24].

Proper utilization of adequately iodized salt was the final target to tackle IDDs, and this means that adequately iodized salt should be added after cooking is finished [16]. Even if availability of adequately iodized salt showed an increment, proper utilization of iodized salt was lower which was 180 (25.7%). It was very low compared to the goal of national coverage of 95% which aimed that adequately iodized salt will be used properly [13]. This indicates that availability and proper utilization of adequately iodized salt should be considered together to prevent IDDs.

There were factors which influence utilization of adequately iodized salt. In line with the study done in the Laelay Maychew district and Iraqi [15, 18], participants who attended formal education were 2 times more likely to use iodized salt properly than those who did not attend formal education. The reason for this might be the educated head of households might have learnt and read about the importance of iodized salt and are practicing it.

Participants who had good knowledge on iodized salt were 3.8 times more likely to use iodized salt properly than those who had poor knowledge on iodized salt. This also showed a similar result with studies done in Gondar, Laelay Maychew, and Pakistan [15, 17, 25]. But the study done in Ghana was on the contrary of the current study that knowledge was relatively high because among most (90.4%) of them, only 64.6% of households exclusively used iodized salt for cooking [19].

This difference might be due to the fact that the present study considers multiple factors, but the previous study identified only one factor. In addition to this, the difference in sociodemographic characters and participant's difference might affect the utilization of iodized salt.

Like a study done in India [24], households who used packed salt were 3 times more likely to use iodized salt properly than those who used nonpacked salt. A study conducted in Poland also supported this finding that iodine losses in the salt tended to increase at high humidity or unlimited access of air [26].

## 6. Limitation of Study

Using only rapid salt-testing kits to determine iodine content was one of the limitations of the study, and this method needs to be supported by the titration method. The study was only a quantitative study, but both qualitative and quantitative studies were needed. Cross-sectional nature of this study does not show casualty of predictors to the outcome variable.

## 7. Conclusions and Recommendations

Knowledge, availability of adequately iodized salt, and utilization of adequately iodized salt remain very low in the district and does not meet the national coverage goal.

Occupational status, educational status, and residency of participants' were identified as predictors associated with knowledge on iodized salt at the household level.

Educational status, knowledge on the use of iodized salt, and packaging were identified as factors associated with the utilization of iodized salt at the household level.

EFMHCACA, ARHB, West Gojjam Zonal health department, and Mecha District heath office should improve availability of adequately iodized salt, and the head of households should be aware of knowledge and utilization of iodized salt by designing proper awareness creation programs.

Government should take measure on companies which produced and distributed inadequately iodized salt to the community.

Health providers should sensitize about the importance of iodized salt and its proper utilization at the household level.

## Figures and Tables

**Table 1:** Sociodemographic characteristics of the head of households in the Mecha District, Western Amhara, Ethiopia, 2018 (*N*=700).

Variables	Category	Frequency	Percentage
Sex of the head of household	Female	639	91.3
	Male	61	8.7

Age	18–24 years	144	20.6
	25–34 years	292	41.7
	35–44 years	153	21.9
	≥45 years	111	15.9

Residence	Rural	610	87.1
	Urban	90	12.9

Educational level	Unable to read and write	443	63.3
	Able to read and write	47	6.7
	Elementary completed	55	7.9
	High school	52	7.4
	College and above	103	14.7

Marital status	Married	517	73.9
	Single	109	15.6
	Others	74	10.6

Monthly income in Ethiopia birr	<500	232	33.1
	500–1000	243	34.7
	≥1000	225	32.1

Current occupation	Housewife	520	74.3
	Merchant	84	12
	Government employee	96	13.7

**Table 2:** Knowledge on iodized salt at the household level in Western Amhara, Ethiopia, 2018 (*N*=700).

Variable	Category	Frequency	Percentage
Knowledge	Poor	499	71.3
	Good	201	28.7

Heard about iodized salt	No	471	67.3
	Yes	229	32.7

Heard from school	No	630	90
	Yes	70	10

Heard from health workers	No	628	89.7
	Yes	72	10.3

Heard from mass media	No	576	82.3
	Yes	124	17.7

Heard from friends or relatives	No	647	92.4
	Yes	53	7.6

Heard from training	No	696	99.4
	Yes	4	0.6

Purchased the salt	Weekly market	37	5.2
	From the shop	643	91.9
	Supermarket	20	2.8

Use cover for their salt container	No	102	14.5
	Yes	598	85.4

Expose salts to sunlight	No	235	33.6
	Yes	465	66.4

Washing the salt with water	No	549	78.4
	Yes	151	21.6

Packaging	No	542	77.4
	Yes	158	22.6

**Table 3:** Availability and utilization of adequately iodized salt at the household level in Western Amhara, Ethiopia, 2018 (*N*=700).

Variable	Category	Frequency	Percentage
Iodization	Adequately iodized (deep blue)	443	63.3
	Slightly iodized (slightly blue)	98	14
	Not iodized (colorless in the kit test)	159	22.7

Utilization	Properly utilized	180	25.7
	Improperly utilized	520	74.3

Know whether the used salt iodized or not	Know	206	29.4
	Do not know	494	70.6

Used packed salt	No	542	77.4
	Yes	158	22.6

Reading pack of salt when purchased	No	543	77.6
	Yes	157	22.4

Store salt in jar	No	102	14.6
	Yes	598	85.4

**Table 4:** Factors associated with knowledge on use of iodized salt at the household level in the Mecha district, Western Amhara, Ethiopia, 2018 (*N*=700).

Variables	Good knowledge (*N*=201)	Poor knowledge ( *N*=499)	COR (95% CI)	AOR (95% CI)	*P* value for AOR
Sex					
** **Male	43	18	7.3 (4.1, 12.9)	1.2 (0.5, 2.6)*∗∗*	*P*=0.74
** **Female	158	481	1.00	—	—

Age					
** **18–24 years	69	75	8.4 (4.1, 16.9)	0.7 (0.3, 1.9)*∗∗*	*P*=0.45
** **25–34 years	98	194	4.6 (2.4, 8.9)	0.9 (0.4, 2.2)*∗∗*	*P*=0.81
** **35–44 years	23	130	1.6 (0.7, 3.4)*∗*	1.2 (0.4, 3.2)*∗∗*	*P*=0.70
** **≥45 years	11	100	1.00	—	—

Educational status					
** **Formal education	176	81	36.3 (22.4, 58.8)	15.0 (9.1, 27.7)	*P* < 0.0001
** **No formal education	25	418	1.00	—	—

Residence					
** **Urban	80	10	32.3 (16, 64)	9.1 (3.6, 22.5)	*P* < 0.0001
** **Rural	121	489	1.00	—	—

Occupation					
** **Government employee	90	6	98 (41, 232)	8.8 (3.1, 24.7)	*P* < 0.0001
** **Merchant	42	42	6.5 (3.9, 10.7)	3.1 (1.6, 5.9)	*P* < 0.0001
** **Housewife	69	451	1.00	—	—

Monthly income in Ethiopia birr					
** **500–1000	32	211	5 (.3,.9)	0.7 (0.4, 1.3)*∗∗*	*P*=0.23
** **≥1000	118	107	3.9 (2.6, 5.9)	1.9 (0.9, 3.6)*∗∗*	*P*=0.06
** **<500	51	181	1.00	—	—

*∗*Not significant in bivariate logistic regression; *∗∗*not significant in multivariate logistic regression.

**Table 5:** Factors associated with utilization of adequately iodized salt at the household level in Mecha district, Western Amhara, Ethiopia, 2018 (*N* = 700).

Variables	Proper utilization (*N*=180)	Improper utilization (*N*=520)	COR (95% CI)	AOR (95% CI)	*P* value for AOR
Sex					
** **Male	33	28	3.9 (2.3, 6.7)	1 (0.5, 2.0)^*∗∗*^	*P*=0.96
** **Female	147	492	1.00	—	—

Age of the head of households					
** **18–24 years	58	86	5.6 (2.8, 11.0)	0.89 (0.5, 1.5)*∗∗*	*P*=0.68
** **25–34 years	85	207	3.4 (1.8, 6.5)	0.8 (0.4, 1.6)*∗∗*	*P*=0.53
** **35–44 years	25	128	1.6 (0.8, 3.4)*∗*	0.6 (0.2, 1.3)*∗∗*	*P*=1.65
** **≥45 years	12	99	1.00	—	—

Knowledge					
** **Good	125	76	13.3 (8.9, 19.8)	3.8 (2.1, 6.8.)	*P* < .0001
** **Poor	55	444	1.00	—	—

Educational level					
** **Formal education	131	126	8.4 (5.7, 12.3)	2.2 (1.2, 3.7)	*P* < .0001
** **No formal education	49	394	1.00	—	—

Residence					
** **Urban	57	33	6.8 (4.3, 10.9)	1.1 (0.6, 2.2)*∗∗*	*P*=0.68
** **Rural	123	487	1.00	—	—

Occupation					
** **Government employee	67	29	11.7 (7.1, 19.0)	1.1 (0.5, 2.6)*∗∗*	*P*=0.76
** **Merchant	27	57	2.4 (1.4, 3.9)	0.7 (0.35, 1.3)*∗∗*	*P*=0.26
** **Housewife	86	434	1.00	—	—

Monthly income in Ethiopia birr					
** **500–1000	37	206	0.6 (0.4, 0.9)	0.7 (0.4, 1.3)*∗∗*	*P*=0.27
** **≥1000	89	136	2.2 (1.4, 3.2)	0.7 (0.4, 1.2)*∗∗*	*P*=0.20
** **<500	54	178	1.00	—	—

Packaging					
** **Yes	106	52	12.9 (8.5, 19.5)	3.3 (1.9, 5.7)	*P* < .0001
** **No	74	468	1.00	—	—

^*∗*^Not significant from the bivariate logistic regression; ^*∗∗*^not significant from the multivariate logistic regression.

## Data Availability

The data used to support the findings of this study are available from the corresponding author upon request.

## Abbreviations

ARHB: Amhara Regional Health Bureau
EBC: Ethiopian Broadcast Corporation
EDHS: Ethiopian Demography and Health Survey
EFMHCACA: Ethiopian Food, Medicine and Health Care Administration and Control Authority
EPHI: Ethiopian Public Health Institute
HHs: Households
ICCIDD: International Council for Control of Iodine Deficiency Disorders
IDD: Iodine deficiency disorders
NIDDCP: National Iodine Deficiency Disorders Control Program
PPM: Parts per million
UNICEF: United Nations Children's Fund
USI: Universal salt iodization
WHO: World Health Organization.
